# Development of a graphene oxide-based assay for the sequence-specific detection of double-stranded DNA molecules

**DOI:** 10.1371/journal.pone.0183952

**Published:** 2017-08-29

**Authors:** Anna Maria Giuliodori, Anna Brandi, Shivaram Kotla, Francesco Perrozzi, Roberto Gunnella, Luca Ottaviano, Roberto Spurio, Attilio Fabbretti

**Affiliations:** 1 School of Biosciences and Veterinary Medicine, University of Camerino, Camerino (MC), Italy; 2 Scuola di Scienze e Tecnologie, Sezione di Fisica, University of Camerino, Camerino (MC), Italy; 3 Dipartimento di Scienze Fisiche e Chimiche (DSFC) Università degli Studi dell'Aquila, L'Aquila, Italy; 4 Istituto CRN-SPIN UOS, L'Aquila, Italy; Helsingin Yliopisto, FINLAND

## Abstract

Graphene oxide (GO) is a promising material for the development of cost-effective detection systems. In this work, we have devised a simple and rapid GO-based method for the sequence-specific identification of DNA molecules generated by PCR amplification. The *csp* genes of *Escherichia coli*, which share a high degree of sequence identity, were selected as paradigm DNA templates. All tested *csp* genes were amplified with unlabelled primers, which can be rapidly removed at the end of the PCR taking advantage of the preferential binding to GO of single-stranded versus duplex DNA molecules. The amplified DNAs (targets) were heat-denatured and hybridized to a fluorescently-labelled single strand oligonucleotide (probe), which recognizes a region of the target DNAs displaying sequence variability. This interaction is extremely specific, taking place with high efficiency only when target and probe show perfect or near perfect matching. Upon GO addition, the unbound fraction of the probe was captured and its fluorescence quenched by the GO’s molecular properties. On the other hand, the probe-target complexes remained in solution and emitted a fluorescent signal whose intensity was related to their degree of complementarity.

## Introduction

The capacity of protecting ourselves from infectious diseases depends, to large extent, on our ability to precisely detect the pathogen in the environment and rapidly activate safeguarding actions and adequate treatments. The detection step often relies on biomolecular approaches that exploit the unique features of the DNA molecule [1]. In addition, these DNA-based methodologies are crucial for the identification of the human genetic variations known to be associated with diseases or that may otherwise predispose to pathological conditions [2]. Nanomaterials have proven particularly helpful for the development of new DNA-based assays that can speed up the procedures and reduce the costs for the detection of pathogens or for the early diagnosis of unfavourable genetic conditions [3,4].

Graphene oxide (GO) is a graphene derivative whose monomolecular sheets are strongly oxidized, with hydroxyl and epoxy functional groups on the basal planes, and carbonyl as well as carboxyl groups at the edges [5]. The presence of these functional groups makes GO strongly hydrophilic and dispersible in water [5].

Several studies demonstrate that both single strand (ss)DNA and RNA can strongly and stably adsorb onto GO due to pi-stacking interactions [6] and, possibly, hydrogen bonding [7] between the nitrogenous bases and the oxidized sheets of GO, whereas dsDNAs and structured RNAs display lower affinities inasmuch less capable of forming the above-mentioned stacking interactions [8]. GO is also a highly efficient quencher of fluorescence with a long-range nanoscale fluorescence resonance energy transfer (FRET) property [9]. The combination of these features makes GO a versatile molecule for the development of novel assays based on fluorescent DNA/RNA detection. In fact, when a single stranded molecule (probe) labelled with a fluorescent dye [6] binds to GO, its fluorescence is quenched by FRET. However, upon addition of a target molecule, which can be a complementary sequence as well as a specific ligand, the interaction with GO is lost and the fluorescence signal is restored [6,10,11].

Thanks to these properties, the GO-mediated fluorescence quenching and recovery of the signal has been used to efficiently detect sequence variations in short ssDNA molecules [6; 10]. However, in its native form DNA is a double-stranded helix, which can also be rather long, a condition that necessarily introduces another layer of complexity in the system. Overall, this complexity is reflected in the various GO-based approaches devised for the sequence-specific detection of short DNA duplexes [12] or native DNA molecules derived from real biological samples [13,14].

In this work, we have developed a simple and rapid GO-based method for the sequence-specific detection of DNA amplicons generated by PCR. To set up this system, we took advantage of the high degree of sequence similarity present among the *csp* genes of the model organism *Escherichia coli* that were amplified by PCR using unlabelled oligonucleotide primers. At the end of the amplification step, the primers can be removed using GO and the target DNAs are heat-denatured and hybridized to a FAM-labelled probe properly designed to recognize a region of the *csp* DNAs displaying discrete values of sequence variability. The interaction between probe and target DNA is extremely specific, taking place with high efficiency only when target and probe show perfect or near perfect complementarity. The unbound fraction of the probe can be readily captured by GO, while the extent of fluorescence emitted by the probe-target hybrids, measured with a simple fluorescence reader, provides a discrimination among variant DNA target sequences.

## Materials and methods

### Buffers

Pfu Buffer (1X): 20 mM Tris-HCl pH 8.3, 10 mM (NH_4_)_2_SO_4_, 10 mM KCl, 1% Triton X-100, 0.1 mg/ml BSA; Taq Buffer (1X): 15 mM Tris-HCl pH 8, 50 mM KCl, 1.5 mM MgCl_2_; GO Buffer (1X): 20 mM Tris-HCl pH 7.4, 100 mM NaCl, 5 mM KCl, 5 mM MgCl_2_.

### Graphene oxide

Commercial GO solution has been purchased from Graphene Supermarket. The GO was characterized by micro-Raman and X-ray photoemission spectroscopy (XPS) and by scanning electron microscopy (SEM). Micro-Raman spectroscopy was performed with a LABRAM spectrometer (Horiba-Jobin Yvon, λ = 633 nm, 1 μm spatial resolution, and 2 cm^−1^ spectral resolution) equipped with a confocal optical microscope. XPS spectra were acquired with a PHI 1257 spectrometer equipped with a monochromatic Al Kα source (hν = 1486.6 eV) with a pass energy of 11.75 eV, corresponding to an overall experimental resolution of 0.25 eV, and were fitted with Voigt line shapes and Shirley backgrounds. The GO solution was spin coated on 100 nm Au(100)/Si in order to perform XPS analysis. SEM images were acquired with a Zeiss-Gemini LEO 1530 system by spin coating the diluted GO solution on 300 nm SiO_2_/Si.

### AFM characterization

AFM was performed in air in tapping mode using a Veeco Digital D5000 system. The samples analyzed with AFM were prepared by drop casting the GO/GO+ssDNA solution on 270 nm SiO_2_/Si substrates.

### PCR amplification

The recombinant plasmids pUT7*cspA* [15], pUT7*cspB*, pUT7*cspC*, pUT7*cspD*, pUT7*cspE* [16], pTZ19*hupA* [17] and pUTZ18*infA* [18] used as templates for the amplification of the genes were already available in the laboratory.

PCR amplifications were carried out in 1X Pfu buffer or 1X Taq Buffer in the presence of 0.2 mM dNTPs, 0.4 μM forward primer, 0.4 μM reverse primers specific for each target gene (see Table 1), and Pfu DNA polymerase or Taq DNA polymerase.

**Table 1:** List of primer sequences.

Primers	Sequences (5′- 3′)	Gene	Amplicon size
**Forward**	ATGTCCGGTAAAATGACTGG	*cspA*	511
**Reverse**	CGGGATCCAAAATCCCCGCCAAATG		
**Forward**	ATGTCCGGTAAAATGACTGG	*cspB*	491
**Reverse**	TCCCAAGCTTTCTTCGTTATCGTATACAG		
**Forward**	ATGTCCGGTAAAATGACTGG	*cspC*	550
**Reverse**	TCCCAAGCTTGTAAAAAGCCTCGCATTCG		
**Forward**	ATGTCCGGTAAAATGACTGG	*cspD*	453
**Reverse**	CGGGATCCATAAAAATGCCAGCCGATC		
**Forward**	ATGTCCGGTAAAATGACTGG	*cspE*	380
**Reverse**	TCCCAAGCTTAAACCCGCTGATTAAGCG		
**Forward**	ATGTCCGGTAAAATGACTGG	*hupA*	606
**Reverse**	CGCGGATCCACGCAGAAAGACAA		

### Purification of amplified DNA fragments

At the end of preparative PCR reactions (≈ 5 ml), DNA was precipitated with 2.5 volumes of absolute ethanol and 1/10 volume of 3M sodium acetate pH 5.2. The precipitated DNA was then recovered by centrifugation at 9 K rpm for 30 min at 4°C (Sorvall SA-600 Rotor). The resulting pellet was rinsed with 5 ml of 70% EtOH, centrifuged again at 9 Krpm for 10 minutes, dried and resuspended in 400 μL of sterile ddH_2_O. Each DNA was further purified from the excess of salts by using the E.Z.N.A PCR clean-up kit and eluted twice using 40 μL of 5 mM Tris-HCl pH 8. The DNA sample volumes were then reduced by Speedvac concentrator (Savant) to achieve a concentration ≥ 300 ng/ μL, as determined by spectrophotometric measurement and agarose gel electrophoresis (1.5%).

### RNA preparation

Full-length mRNAs were obtained by *in vitro* transcription with T7 RNA polymerase and purified as described [19]. The DNA templates used for the transcription reactions were the same DNA amplicons described above.

### Spectrofluorimeter measurements

The carboxyfluorescein (FAM)-labeled single-stranded probe (FAM-P: 5’-FAM-CTGGATAGCGGAGAAGTG-3’) was hybridized with increasing amounts of the complementary target oligo (T-Oligo: 5’-CACTTCTCCGCTATCCAG-3’) in 1X GO buffer for 10 min at 20°C, before addition of 15 μg/ml of GO. After 10 min incubation at room temperature, fluorescence spectra were recorded in the 510–600 nm range using an F-4500 Fluorescence Spectrophotometer (Hitachi).

### GO assays for the detection of FAM-P+Target DNA complexes

In a typical reaction mixture (24 μL), FAM-P (75 nM) was incubated at 95°C for 3 min (ThermoStat Plus–Eppendorf incubator) in 1X GO buffer with the indicated amounts of either the complementary T-Oligo or the *csp* target DNAs. Samples were then cooled down to 25°C (10°C/min) and spun before the addition of 1 μL of GO (final conc. 8 μg/ml). When hybridization was carried out at higher DNA concentrations, the reaction was performed in 4 μL containing 1X GO buffer, 0.45 μM FAM-P and either 0.45 μM T-Oligo or 0.3 μM target DNAs. Samples were denatured at 95°C for 1 min, left on ice for 1 min, and incubated at 20°C for 15 min before addition of 21 μL of GO in 1X GO buffer at a final conc. of 8 μg/ml.

After GO mixing, samples were incubated at 25°C for 10 min and then transferred to a Black and White Wallac plate. Fluorescence was measured in an FLUOstar Omega instrument with a gain of 800, excitation 485 nm and emission 520 nm.

### GO assay in the presence of dNTPs or PCR primers

The effect of PCR primers on the GO-based detection system was tested in 24 μL reaction by mixing increasing amounts of these components with 75 nM FAM-P and 150 nM T-Oligo in 1X GO buffer. The effect of dNTPs on the same GO-based system was tested in 24 μL reaction by mixing increasing amounts of T-Oligo with 75 nM FAM-P in the presence or in the absence of 0.2 mM dNTPs in 1X GO buffer. Samples were denatured, hybridized, mixed with GO and read in the FLUOstar Omega as described above.

### GO assay under PCR conditions (post-PCR primers removal)

FAM-P (final conc. 75 nM) and GO buffer (final conc. 1X) were added to a reaction mixture containing 1X Taq Buffer, 1.5 mM MgCl_2_, 0.2 mM dNTPs, 0.02U/μL Taq DNA Polymerase, and 37 nM target DNAs. A control curve was prepared under the same conditions mixing FAM-P with increasing amounts of T-Oligo. Samples were denatured, hybridized, mixed with GO and read in the FLUOstar Omega as described above.

### GO assay for the detection of FAM-P+Target RNA complexes

The hybridization reaction was performed in 9 μL containing 1X GO buffer, 2 μM FAM-P and either 20 μM T-Oligo or 8 μM target RNAs. Samples were denatured at 95°C for 1 min, left on ice for 1 min, and incubated at 20°C for 30 min before the addition of 262 μL 1X GO buffer. After 5 min on ice, 24 μL of each reaction mixture were transferred to a new tube and incubated at 20°C for 15 min with GO (final conc. 8 μg/ml). The Fluorescence was measured as described above using a Black and White Wallac plate.

### Primer removal

Each sample (48 μL) contained 1X GO buffer, 1X Taq Buffer, 1.5 mM MgCl_2_, 0.2 mM dNTPs, 0.02U/μL Taq DNA Polymerase, 9 ng/μL *cspC* DNA, 1 μM of FAM-labeled oligonucleotide (5’-FAM- ATGTCCGGTAAAATGACTGG-3) and the increasing amounts of GO indicated in the figure. After 10 min incubation at 20°C, 10 μL of each sample were used to measure the fluorescence in the FLUOstar Omega. The remaining volume was subjected to centrifugation at 10 K rpm for 4 min (or 2 K rpm for 3 min) at room temperature to pellet down GO and the GO-bound molecules. The upper portion of each supernatant (20 μL) was split into two aliquots (10 μL). One aliquot was subjected to 1.5% agarose gel electrophoresis followed by ethidium bromide staining and band visualization under UV light. The other aliquot was used for Fluorescence measurement in FLUOstar Omega.

## Results

### GO characterization

The unambiguous identification of GO was performed with micro-RAMAN spectroscopy. The GO Raman spectrum with signature-like spectral feature of the D and G peaks at 1330 and 1600 cm^-1^, respectively, is shown in S1 Fig. The position, width and relative intensity of these peaks are in agreement with those reported in literature [20].

The chemical composition of the GO used in the experiments was investigated by means of XPS (S2 Fig). The quantitative analysis of the XPS spectra shows a C/O ratio of 2, in line with the values reported in literature [21]. This oxidation degree explains the good solubility in water of the GO, which is a prerequisite of the methodology of this work. In addition, S3 Fig shows a SEM image of a spin coated sample of the GO solution. The images demonstrate that GO is perfectly exfoliated into single layer flakes having a size in the range of 0.2–2 μm. The GO flakes size is by far large enough to accommodate the ssDNA probe having an estimated total length of about 10 nm in the most extended form.

### Sequence-specific detection of dsDNA and RNA using GO

The polymerase chain reaction (PCR) is a technique which entails an enzymatic DNA amplification to generate thousands to millions of copies of a selected DNA region starting from a few DNA molecules [22]. To set up a GO-based method for the sequence-specific detection of DNA molecules generated by PCR, we amplified five out of the nine *csp* genes of *E*. *coli* (*cspA* to *cspI*), which are all monocistronic, similar in size and evolutionarily conserved [23].

**Fig 1:**
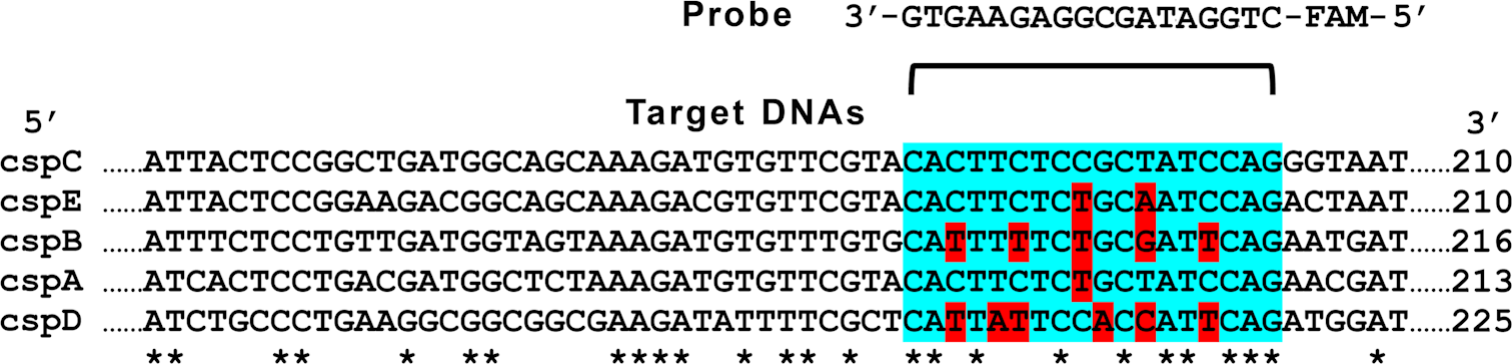
Sequences of target DNAs and probe. Nucleotide sequence alignment of *cspC* (eco:b1823, KEEG), *cspE* (eco:b0623, KEEG), *cspB* (eco:b1557, KEEG), *cspA* (eco:b3556, KEEG), and *cspD* (eco:b0880, KEGG). Identical residues are indicated by asterisks. Dots indicate the missing bases of the protein coding sequences. The single strand oligonucleotide (18-mer) conjugated with the fluorescent dye carboxyfluorescein (FAM) used as a probe in this work is fully complementary to the region of *cspC* indicated in cyano, while having an increasing number of mismatches (in red) with the other *csp* shown in the alignment.

The strategy of the present work relies on the hybridization of these target *csp* DNA amplicons with a chemically synthesized 18mer oligonucleotide conjugated with a fluorescent dye (FAM) (5’-FAM-CTGGATAGCGGAGAAGTG-3’). The sequence of this probe (FAM-P) matches perfectly a short region of the *cspC* gene while having an increasing number of mismatches with the other *csp* genes analyzed in this work (Fig 1).

**Fig 2:**
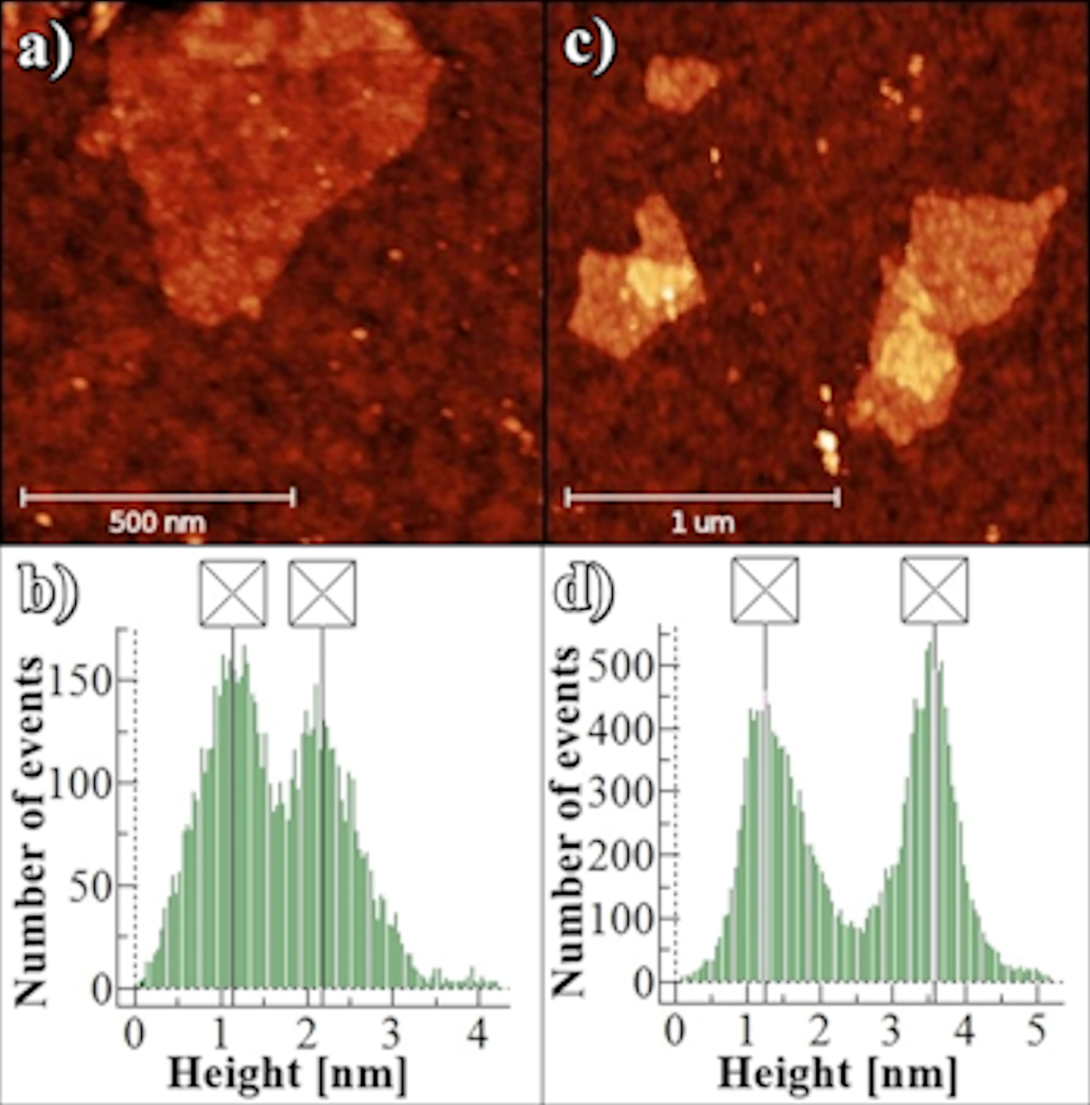
AFM height images. (A) single layer GO sheets deposited on SiO_2_ substrates; (C) FAM-P + single-layer GO sheets complex; (B) and (D) histogram analysis of (A) and (C), respectively.

The morphology of GO and GO + FAM-P complex was measured by AFM. Typical AFM images of dispersed single layer GO and GO + FAM-P complex (deposited, in this case by drop casting on SiO_2_) are shown in Fig 2A and 2C. The measured thickness of a single layer of GO flake typically ranges between 1.0 to 1.4 nm [5,24]. The thickness value changes with the AFM settings parameters [25]. In our studies, the thickness of a single layer GO sheet (Fig 2B) is about 1.08 nm. Fig 2C shows the typical AFM image of the FAM-P+GO complex, where the bright areas on the GO surface might be due to the adsorption of ssDNA. In this case, the thickness of the complex is about 2.54 nm (Fig 2D).

**Fig 3:**
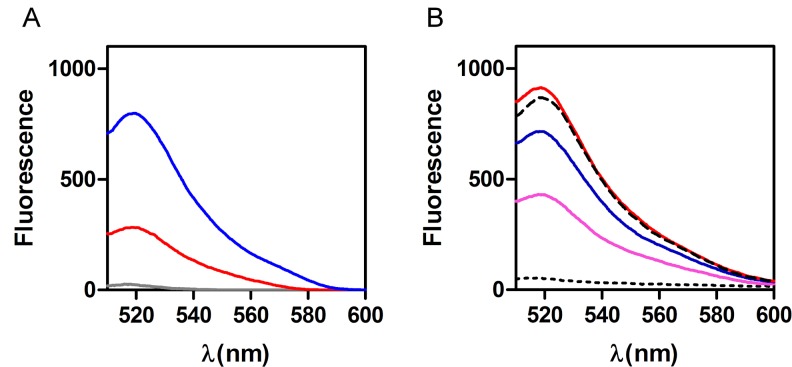
Fluorescence emission spectra of FAM-P. (A) 10 nM (gray), 50 nM (red) and 100 nM (blue) of FAM-P in GO Buffer. (B) FAM-P (100 nM) emission after pre-incubation with 0 nM (dotted line), 200 nM (magenta), 400 nM (blue), or 800 nM (red) of complementary target oligonucleotide (T-Oligo: 5’-CACTTCTCCGCTATCCAG-3’) and mixing with 15 μg/ml of GO. The broken line indicates the control spectrum obtained with 100 nM FAM-P alone. Further details are given in Materials and Methods. The raw data of these experiments are shown in S1 Dataset.

The fluorescence emission of the dye is maximal at about 520 nm and depends on the concentration of FAM-P in solution (Fig 3A). When FAM-P is mixed with GO, its fluorescence is quenched (Fig 3B) while the incubation with a complementary target oligonucleotide (T-Oligo: 5’-CACTTCTCCGCTATCCAG-3’) before GO addition produces a partial recovery of fluorescence whose extent is proportional to the concentration of the target T-Oligo (Fig 3B).

**Fig 4:**
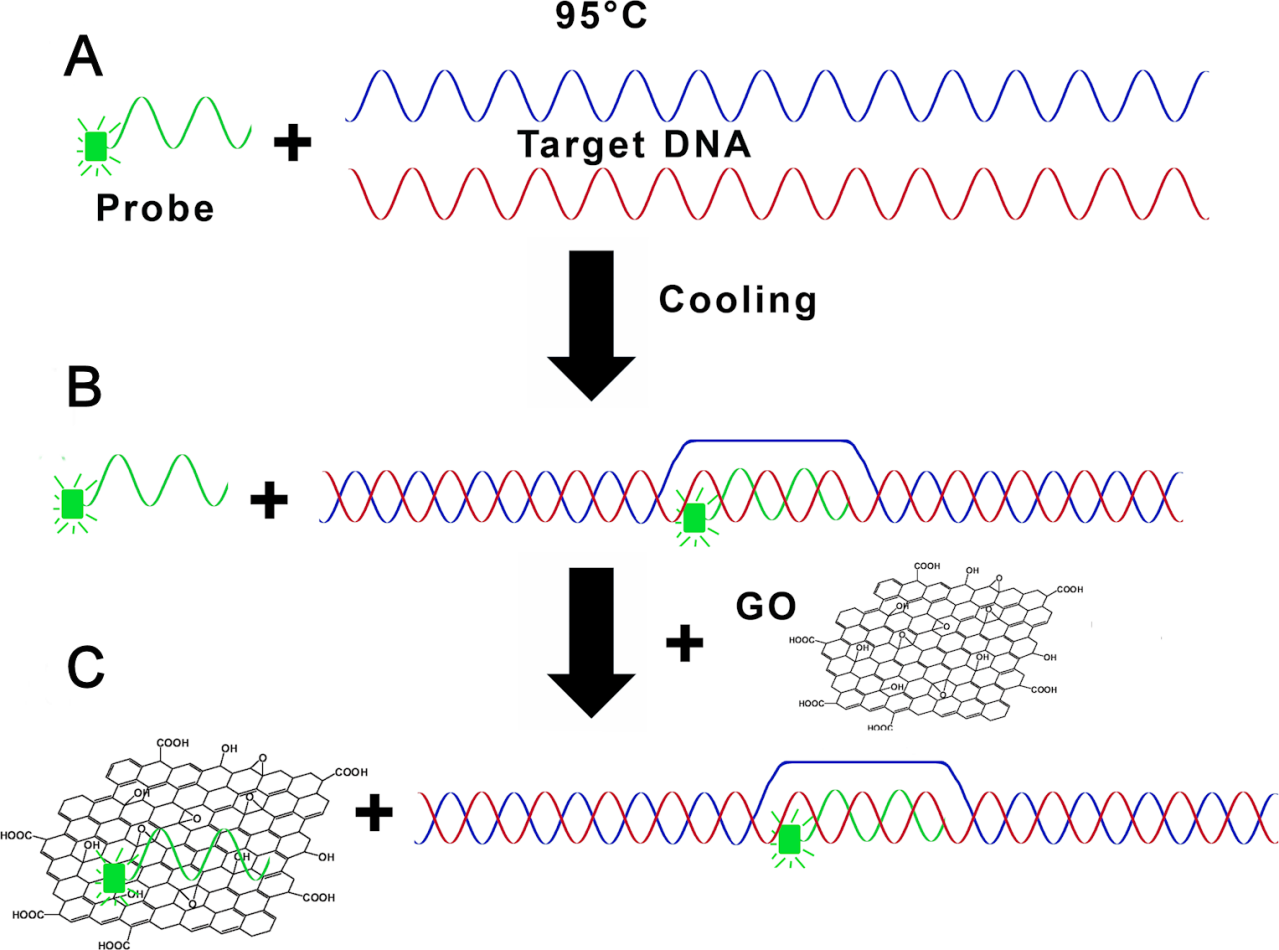
Schematic description of the GO-based system devised for the sequence-specific detection of PCR products. (A) Probe (FAM-P) and target dsDNA are mixed and denatured at 95°C; (B) after cooling, Target and FAM-P base pairs and the original DNA duplex renatures with the exception of the positions hybridized with (and near) the Probe; (C) adsorption of free FAM-P onto GO results in fluorescence quenching; the Target-FAM-P complexes that do not bind GO emit a fluorescent signal.

Unlike the straightforward interaction between the single stranded FAM-P and T-Oligo, the interaction of FAM-P with the amplified *csp* genes requires the preliminary separation (denaturation) of the two strands that form the DNA duplex (Fig 4A). To induce this denaturation, target *csp* DNA and FAM-P are mixed and heated to 95°C. The subsequent cooling enables FAM-P to base pair with the complementary sequence (if present) in the target DNA, locally displacing the original strand. Eventually, the original DNA strands renature with the exception of the positions hybridized with (and near) the probe (Fig 4B). Evidently, the binding of the probe to the target (hybridization) is in competition with the re-formation of the original DNA duplex (renaturation), therefore the probability of hybridization winning the competition depends on the kinetics of the reaction, which in turn is affected by the degree of matching between the molecules [26]. Upon addition of GO to this mixture, unbound FAM-P and, possibly, partially renaturated duplexes, will bind to GO. On the contrary, DNA duplexes and the complex *csp* DNA+FAM-P are expected to remain free due to their double-stranded conformation (Fig 4C). Since GO quenches only the fluorescence of the bound molecules, the unquenched fluorescence should represent the amount of these free *csp* DNA+FAM-P complexes.

**Fig 5:**
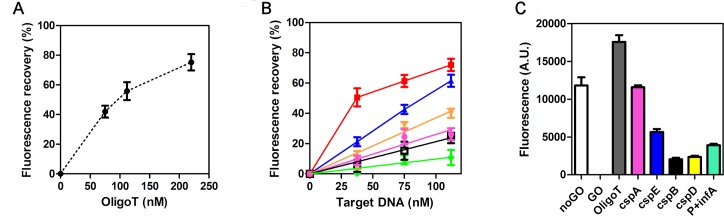
GO assays for the detection of FAM-P+Target DNA/RNA complexes. Relative fluorescence of FAM-P (75 nM) after incubation with the indicated concentrations of T-Oligo (A) or Target DNAs (B) and subsequent addition of GO (8 μg/ml). The target DNAs are: *cspC* (red square), *cspE* (blue triangle), *cspD* (orange reversed triangle), *cspA* (magenta circle), *cspB* (open square) and *hupA* (green diamond). (C) Fluorescence emission observed after incubation of FAM-P (2 μM) with T-Oligo (20 μM) or the indicated mRNAs (8 μM) followed by dilution and GO addition (8 μg/ml). Further details are given in the text and in Material and Methods. The data points are the average of two experimental samples; error bars represent the standard deviations. The signal measured with FAM-P +GO was taken as background and subtracted from each experimental point. The percentage is calculated taking F-F_GO_ as 100%, where F and F_GO_ are the fluorescences measured with FAM-P alone and FAM-P +GO, respectively. The raw data of these experiments are shown in S2 Dataset. The replicates of independent experiments reported in Panels A and B, are shown in S5 Fig.

To prove the feasibility of this reaction, we purified the *csp* DNAs prepared by PCR and carefully determined their concentration. Subsequently, we hybridized these target DNAs with FAM-P and then we added GO. To work with small amounts of input DNA, we optimized the reaction volume to 25 μL and measured the fluorescence in microtiter plates using the FLUOstar Omega instrument at fixed excitation and emission wavelengths (485 nm and 520 nm, respectively). Under these conditions, the emission of 75 nM of FAM-P is well quenched by 8 μg/ml of GO (S4 Fig) and its recovery by the complementary T-Oligo is comparable to that measured at the spectrofluorimeter (Fig 5A). As shown in S4 Fig, a higher amount of GO switches off the FAM-P+T-Oligo signal, probably because under this condition also the complex is captured by GO and its fluorescence is thus quenched. In fact, although dsDNA displays low affinity for GO, its adsorption onto GO can take place when the concentration of either one or the other reacting species is increased above a certain level, as demonstrated by Park et al. [27].

With the amplified target DNAs, we observed a fluorescence emission which increased with increasing matching between FAM-P and targets (Fig 5B). In particular, at the lowest target DNA concentration (37.5 nM) the perfectly matching *cspC* DNA induced a fluorescence emission corresponding to ≈50% of the fluorescence measured in the absence of GO (initial fluorescence) and fully comparable to that observed with the T-Oligo (Fig 5A). On the contrary, the presence of two mismatches (*cspE*) elicited an emission that was only ≈20% of the initial fluorescence signal and the values further decreased (≤ 10%) with the other DNA targets containing increasingly more mismatches (3–6). As a control, we performed the same experiment using a completely unrelated amplicon which resulted from the DNA amplification of *hupA*, the gene encoding the alpha subunit of nucleoid protein HU [17] whose size is comparable to that of *csp* genes. As expected, very little fluorescence emission was observed with this negative control. Notably, when the concentration of the unrelated dsDNA was raised above 200 nM, a non-specific release of FAM-P from GO was observed (not shown). This is likely due to the adsorption of a fraction of this dsDNA onto GO and the consequent non-specific detachment of the bound FAM-P, in agreement with the results of Park et al. [27].

To our surprise, we could not detect any fluorescence with the one mismatched *cspA* DNA (Fig 5B), even when the hybridization reaction between FAM-P and target DNAs was carried out at much higher concentration of reactants (300 nM and 450 nM, respectively; see S6 Fig) before dilution and interaction with GO.

To understand if this unexpected result could be attributed to an intrinsic incapacity of the probe to recognize its complementary sequence within the *cspA* context, we produced by *in vitro* transcription the RNA counterpart of *csp* genes, their messenger RNA (mRNA). The *cspA* mRNA was initially used to set up the conditions of the GO-RNA detection system, along with the unrelated *infA* mRNA [18] used as a control. Unlike the DNA detection, when hybridization was carried out at low concentration of reagents (75 nM FAM-P and 20–30 nM of target mRNAs) the measured fluorescence was independent of the matching between probe and target (S7A Fig). However, when the concentration of the interacting molecules was considerably raised and the target mRNA/FAM-P molar ratio was brought to 4, FAM-P was perfectly capable of detecting the targets with the expected selectivity (Fig 5C and S7B Fig). In fact, using this stoichiometric ratio of reagents, the best fluorescent signal was obtained with *cspA* mRNA (1 mismatch), followed in decreasing order by *cspE* mRNA (2 mismatches), *cspD* and *cspB* mRNAs (6 and 5 mismatches, respectively) and the unrelated *infA* mRNA (Fig 5C). However, it is worth mentioning that the use of GO for the sequence-specific detection of RNAs extracted from cells is impractical under the tested conditions because it requires very high amounts of RNA target.

### GO and PCR

**Fig 6:**
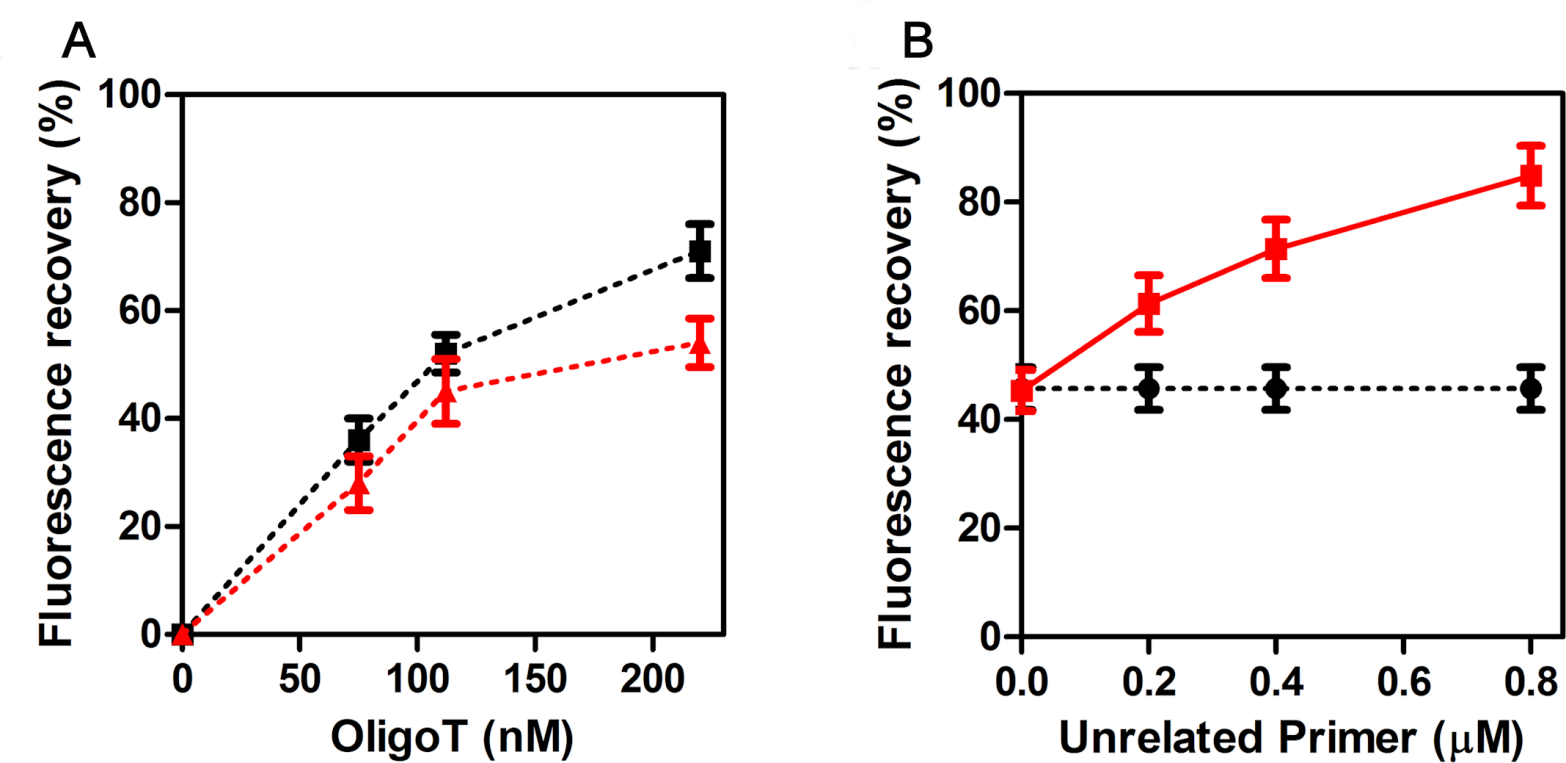
Effects of dNTPs and PCR primers on the GO-based assay. FAM-P (75 nM) was incubated: (A) with the indicated concentration of T-Oligo in the presence (red triangle) or in the absence (black square) of 200 μM dNTPs; (B) with 120 nM of T-Oligo in the presence (red square) or in the absence (black circle) of the indicated concentration of PCR primers. Fluorescence was measured after mixing the samples with 8 μg/ml GO. Further details are given in the text and in Material and Methods. The data points are the average of two experimental samples; error bars represent the standard deviations. The signal measured with FAM-P +GO was taken as background and subtracted from each experimental point. The percentage is calculated taking F-F_GO_ as 100%, where F and F_GO_ are the fluorescences measured with FAM-P alone and FAM-P +GO, respectively. The raw data of these experiments are shown in S3 Dataset.

In the previous section, we showed how simple and straightforward is to perform a GO-based reaction for the discrimination of small sequence variations in amplified DNA molecules subjected to a clean-up step to remove unincorporated primers and deoxynucleotides (dNTPs). However, this clean-up step is time-consuming and causes a certain loss of DNA sample. Therefore, we investigated whether the reaction with GO could be performed directly at the end of the PCR, in the presence of both unincorporated primers and dNTPs. To answer this question, we initially tested if these molecules could influence the interaction of GO with the duplex formed between FAM-P and the complementary T-Oligo. As can be seen in Fig 6A, dNTPs slightly affect the fluorescence emission observed with FAM-P, T-Oligo and GO. On the other hand, increasing amounts of PCR primers, in the concentration range expected in a PCR, caused an increase in fluorescence that is fully not specific (Fig 6B). Therefore, we verified if the PCR primers could be removed taking advantage of the GO’s propensity to bind short ssDNA molecules.

**Fig 7:**
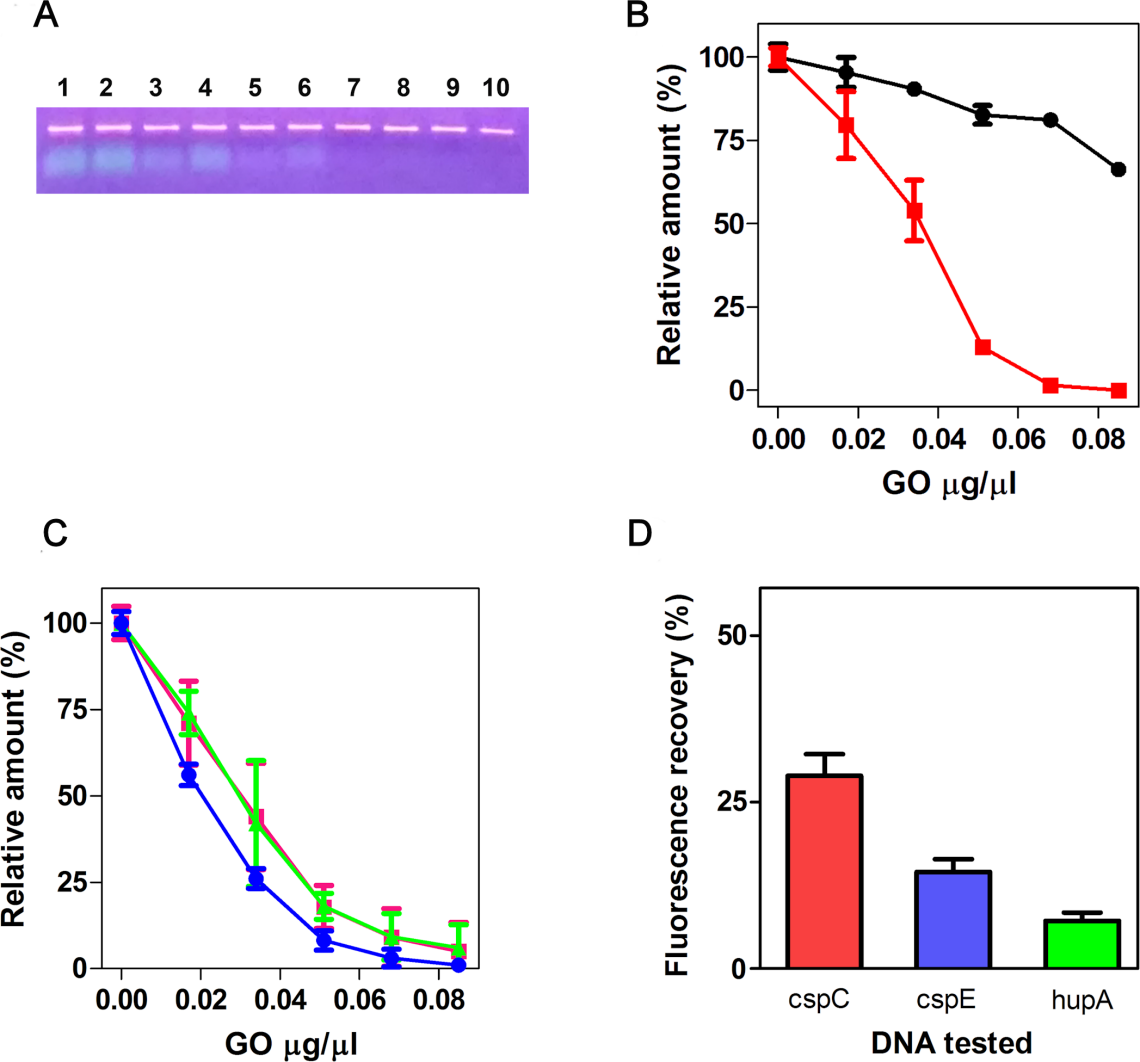
GO-based removal of PCR primer and sequence-specific amplicon detection. (A) The DNA samples prepared as described in the text were incubated with the amounts of GO indicated below. After centrifugation, an aliquot of each supernatant was subjected to 1.5% agarose gel electrophoresis followed by ethidium bromide staining and band visualization under UV light. Orange and green bands correspond to *cspC* DNA and fluorescent primer, respectively. Lanes 1–2: no GO; lanes 3–4: 0.018 mg/ml GO; lanes 5–6: 0.032 mg/ml GO; lanes 7–8: 0.05 mg/ml GO; lane 9: 0.068 mg/ml GO; lane 10: 0.086 mg/ml GO (B) Quantification by densitometry of the bands shown in (A); black circles: *cspC* DNA; red squares: fluorescent primer. The percentage was calculated taking the average band intensities of lanes 1 and 2 as 100%. (C) Relative Fluorescence emitted by the samples incubated with the indicated amounts of GO (blue) or by the supernatants of the same samples recovered after centrifugation at 10 K rpm (pink) or 2 K rpm (green). (D) FAM-P (75 nM) was incubated with the indicated Target DNAs in the presence of Taq Buffer, dNTPs and Taq DNA Polymerase at the concentrations indicated in Materials and Methods. Fluorescence was measured after GO addition.

To perform this analysis, we mixed a fluorescent primer with a solution containing the Taq DNA polymerase, its reaction buffer, 1X GO buffer, dNTPs, and the amplified *cspC* DNA (see M&M) at the typical concentrations attained in a PCR. This mixture was incubated with increasing amounts of GO and then divided into two parts, one subjected to centrifugation and the other left untreated. During the centrifugation, GO and the bound molecules sediment at the bottom of the tubes forming a pellet, while free DNA and free primers remain in the supernatant. Aliquots of the supernatants were withdrawn and subjected to agarose gel electrophoresis to separate the *cspC* DNA from the fluorescent primer (Fig 7A). The densitometric quantification of the corresponding bands is shown in Fig 7B. Unequivocally, the GO treatment drastically sequesters the primers, while marginally affecting the dsDNA. In fact, a GO concentration of 0.07 mg/ml removes almost all primer with a loss of amplified DNA of about 20–25%. This result is confirmed by the fluorescence of both supernatants (Fig 7C, green and magenta lines) and untreated samples (Fig 7C, blue line), this latter containing the primer in its free (fluorescent) and GO-bound (quenched) form. From the comparison of these two types of samples, it can also be deduced that the centrifugation step causes a certain detachment of the primer from GO, even at a reduced speed (Fig 7C, green line). The GO’s ability of binding and holding the primer is also strongly affected by the composition of the buffer used for the PCR. In our experience, the buffer containing BSA should be avoided insofar as this protein interferes with the DNA binding capacity of GO. Concerning the dNTPs, they presumably do not bind GO under the tested conditions, as seen for the ATP and the corresponding nucleoside Adenosine (S9 Fig). Nevertheless, they do not interfere with the subsequent reactions (see above). Finally, the result shown in Fig 7D demonstrates that, in the absence of the PCR primers, the sequential incubation of the PCR mixtures with FAM-P and GO leads to a specific recognition of the DNA targets. In fact, the complementary *cspC* DNA generates a fluorescence value that is twice as much that generated with the 2-mismatched *cspE* DNA and 4-fold that observed with the unrelated *hupA* DNA. This result is fully comparable to that previously observed with the totally cleaned-up DNA targets (Fig 5B).

The data points are the average of two experimental samples; error bars represent the standard deviations. In (D) the signal measured with FAM-P +GO was taken as background and subtracted from each experimental point. The percentage was calculated taking F-F_GO_ as 100%, where F and F_GO_ are the fluorescences measured with FAM-P alone and FAM-P +GO, respectively. The raw data of these experiments are shown in S4 Dataset. The replicate of the experiment of Panels C, is shown in S8 Fig.

## Discussion

We have developed a simple GO-based method for the sequence-specific detection of dsDNA using a FAM-labelled ssDNA probe and long denatured dsDNA targets. Precise recognition of few base mismatches in DNA sequences with high sequence similarity is important because it provides information on genetic mutations and variations. Our system offers many advantages. First of all, the detection can be carried out directly in the small volumes (25–30 μl) of an analytical PCR mixture and with the amounts of DNA generally obtained at the end of this small-scale reaction, which correspond to about 8–12 ng DNA/μl for bacterial amplicons of 400–500 bp. At the end of the reaction, the unincorporated primers can be removed with a short incubation with GO followed by a brief centrifugation. The little loss of amplified DNA recorded at this stage does not affect the next step, because even a relatively small DNA amount (≤ 37.5 nM) is sufficient to elicit a sequence-specific signal, as shown in Fig 5B. At these target concentrations, which correspond to 11 ng/μL for molecules of an average length of 450 bp as those used here, a small amount of fluorescent FAM-labelled probe (1.87 pmoles) is sufficient to generate a good signal in the presence of the perfectly matching DNA. This greatly reduces the experimental costs, mainly in comparison to other detection systems based on fluorescent reading such as Real Time-PCR. In fact, despite the unquestionable benefits and the numerous applications of this technique, the elevated cost of both the instrument and the reaction, which is about three-fold that of a conventional PCR [28], can be an insurmountable obstacle for laboratory settings with restricted resources.

As described above, our GO-based approach relies on a series of simple yet reliable steps whose correct combination leads to reproducible and sound results. None of these steps, *per se*, is original, but the manner in which they are sequentially combined represents a new and streamlined procedure in the diagnostic field.

In the first action, that is the DNA amplification by means of PCR, our system allows the use of the same pair of primers for the amplification of homologous target genes, as the recognition with the sequence-specific probe occurs in a subsequent step, unlike other GO-based approaches [13,14]. These “universal” primers can be designed to anneal in the most conserved regions of evolutionary related genes commonly used for bacterial identification, such as 16S rDNA, *rpoB*, *gyrB*, *tuf*, *etc*. [1], whereas the fluorescent probe can be devised to recognize the intervening variable regions. The use of a common pair of primers is particularly valuable because, under optimized conditions, ensures the synthesis of comparable DNA amplicons, in terms of both size and amount, even when the amplification is accomplished with different DNA templates.

An additional advantage of our system is that the detection takes place in a step subsequent to the DNA amplification. When the PCR itself is used as a diagnostic tool, the absence of DNA amplification is taken as proof of the absence of the template DNA. Nevertheless, the possibility that the lack of signal is due to malfunctioning of the reaction cannot be ruled out, even when all controls are correctly carried out. On the contrary, when PCR and detection are kept separated, the presence of amplification products provides evidence that the PCR was succesfully completed for all samples and the operator can proceed to perform the detection step. Most of detection systems rely on an oligonucleotide probe with a radioactive, chemiluminescent or fluorescent moiety, whose hybridization with a target DNA produces a specific signal. Either the probes or the targets are normally bound to solid supports (membranes or slides) so that clean up steps can be carried out to wash away the unbound materials. The use of GO simplifies the detection, insofar as it eliminates the need of both the binding of nucleic acids to a solid support and the subsequent washes: probe-target interactions occur in solution and the washing step is avoided thanks to the GO’s quenching properties.

In the course of our study, we have verified that unexpected factors unrelated to the matching of target and probe can influence the GO–based detection, as in the case of *cspA* DNA. The lack of signal between *cspA* DNA and FAM-P can be explained by two possible events. In the first case, the FAM-P/*cspA* hybrid forms but remains close to the GO surface and its fluorescence is quenched by the GO’s FRET. The second possibility is that, for kinetics reasons, the renaturation reaction prevails over the probe-target hybridization. The lack of signal is not necessarily a negative event, as it could be exploited to stress the difference between perfectly matching and nearly-matching DNA targets, as for *cspC* and *cspA*, whose fluorescence signal would be otherwise very similar. However, it could have also some drawbacks, as demonstrated by the results of the experiments that we have conducted using *csp* DNAs of about 200 bp (not shown). Unexpectedly, we have obtained very low fluorescence signals with these shorter DNA targets, even with the perfectly matching *cspC*. We believe that also this behaviour could be ascribed to the GO quenching of partially formed duplexes or to unfavourable hybridization kinetics. Therefore, we recommend to use targets DNA with a size of 400–600 bp, as those used in this work, and to select the fluorescent probe displaying the best performance in terms of target discrimination.

The hybridization between target and probe can be affected by diverse factors, among which the probe length. In fact, for opposite reasons, too short or too long oligonucleotide probes can give rise to spurious hybridization. To increase the target-probe specificity and selectivity, the concept of a split-oligonucleotide system (split-probes) has been introduced [2]. This system consists of two (or more) probes able to bind the target to adjacent positions. Thanks to cooperative interactions, the binding affinities and the recognition capacity of the probes for the target are significantly improved. In light of these considerations, we believe that also our GO-based detection system could be enhanced by designing suitable split-probes.

## Conclusion

Food-borne diseases and microbial infections are increasingly becoming a critical health problem worldwide. Many different molecular tools for microbial detection and diagnostics are available, such as PCR, multiplex PCR, real-time PCR, microarrays, molecular typing and next-generation sequencing [1, 29], each with its own advantages and drawbacks. Indeed, many of these systems are intrinsically time-consuming or require expensive instrumentations, dedicated facilities or highly qualified personnel. The possibility of developing in the near future new detection systems based on nanomaterials will be critical for reducing both time and costs of these analyses. The use of nanomaterials in the biological field has indeed paved the way to the development of new biosensors [4]. Some of these biosensors are based on colorimetric reactions that relay on the aggregative properties of the nanomaterials, as in the case of the gold nanoparticles [8], or in their catalytic activities, as for the carbon nanotubes. These assays display different degrees of complexity and overall a good (in some cases excellent) sensitivity for the target. However, in our opinion GO is the most promising nanomaterial for the development of DNA-based tests which can conjugate sensitivity, simplicity and low costs. Accordingly, the system described in this work requires a standard equipment available also in small laboratories, the use of standard DNA probes much less expensive than those based on modified nucleic acid such as PNA [12] and short experimental times.

In conclusion, we believe that the application presented here could be used in the future to build GO-based macroarrays ensuring a simple yet unequivocal identification of disease-causing microorganisms. Initially, bacteria will be isolated from a biological sample. Next, a set of target gene regions will be amplified from the isolated bacteria by PCR. The amplified DNAs will correspond to gene regions commonly used for bacterial identification (see above). Subsequently, the target DNAs will be simultaneously tested in solution in the macroarrays exploiting the distinctive features of GO, using a wide range of fluorescent probes capable of recognizing with high specificity the sequences of the above mentioned key genes of selected pathogenic bacteria. The presence of fluorescent signals will indicate that the original specimen contained the pathogens whose DNA sequences were used as probes in the macroarray.

## Supporting information

S1 Fig Raman spectrum of GO. The GO Raman spectrum is characterized by the D and G band positioned respectively at 1330 and 1600 cm^-1^ and with a full width at half maximum of 100 and 74 cm^-1^. The relative intensity I_D_/I_G_ is equal approximately to 1.4.
(JPG)

S2 Fig C 1s XPS spectrum of GO film deposited on 100 nm Au/Si. The C 1s spectrum is fitted by the sum of three components assigned to C atoms belonging to: aromatic rings and hydrogenated carbon (C = C/C-C, C-H, 284.9 eV), hydroxyl groups and epoxy groups (C-OH, C-O-C, 286.9 eV), carbonyl groups (C = O, 288.0) and carboxyl groups (C = O(OH), 289.0 eV). The relative weight of each component is equal to 47%, 44%, 5% and 4%, respectively, while the overall C/O ratio is ≈2.
(JPG)

S3 Fig SEM image of GO flakes deposited by spin coating on 300 nm SiO_2_/Si. The image shows that the GO flakes size ranges between 0.2 and 2 μm.
(JPG)

S4 Fig Fluorescence emission of FAM-P (75 nM) pre-incubated with 225 nM of T-Oligo before GO addition (blue) or directly mixed with GO (red). Further details are given in the text. The data points are the average of two experimental samples; error bars represent the standard deviations. The signal measured in the absence of the fluorophore was taken as background and subtracted from each experimental point. The percentage was calculated taking F-F_0_ as 100%, where F and F_0_ are the fluorescences measured in the presence and in the absence of the fluorescent primer, respectively.
(JPG)

S5 Fig Replicate of GO assays for the detection of FAM-P+Target DNA complexes. Relative fluorescence of FAM-P (75 nM) after incubation with the indicated concentrations of T-Oligo (A) or Target DNAs (B) and subsequent addition of GO (8 μg/ml). The target DNAs are: *cspC* (red square), *cspE* (blue triangle), *cspA* (magenta circle), *cspB* (open square) and *hupA* (green diamond). Further details are given in the text and in Materials and Methods. The signal measured with FAM-P +GO was taken as background and subtracted from each experimental point. The percentage is calculated taking F-F_GO_ as 100%, where F and F_GO_ are the fluorescences measured with FAM-P alone and FAM-P +GO, respectively.
(TIF)

S6 Fig GO assays for the detection of FAM-P+Target DNA complexes. Absolute (A) or Relative (B) fluorescence of FAM-P (450 nM) after incubation with T-Oligo (450 nM) or the indicated Target DNAs (300 nM), and subsequent addition of GO (8 μg/ml). Further details are given in Material and Methods. The florescence measured with FAM-P +GO was taken as the background and subtracted from each experimental point. In (b), the percentage is calculated taking F-F_GO_ as 100%, where F and F_GO_ are the florescence measured with FAM-P alone and FAM-P +GO, respectively.
(JPG)

S7 Fig GO assays for the detection of FAM-P+RNA complexes. FAM-P at the concentration of (A) 75 nM or (B) 3 μM was incubated with the indicated concentration of *cspA* (magenta) or *infA* (cyano) mRNAs. The fluorescence was recorded after GO addition as described in Materials and Methods. The signal measured with FAM-P +GO was taken as background and subtracted from each experimental point. The percentage is calculated taking F-F_GO_ as 100%, where F and F_GO_ are the florescence measured with FAM-P alone and FAM-P +GO, respectively.
(JPG)

S8 Fig Replicate of GO-based removal of PCR primer. A fluorescent primer (24 pmoles) was incubated with the amounts of GO indicated in the figure under the conditions described in Materials and Methods. After centrifugation at 10 Krpm for 5 minutes, the fluorescence of 10 μL supernatants was read using the FLUOstar Omega instrument. The data points are the average of two experimental samples; error bars represent the standard deviations. The signal measured in the absence of the fluorophore was taken as background and subtracted from each experimental point. The percentage was calculated taking F-F_0_ as 100%, where F and F_0_ are the fluorescences measured in the presence and in the absence of the fluorescent primer, respectively.
(JPG)

S9 Fig ATP and Adenosine binding to GO. The GO amounts indicated in the figure were incubated in 1X GO buffer with (A) 60 μM and (B) 120 μM ATP or (C) 5 μM and (D) 160 μM Adenosine in 500 μL of reaction volume for 10 min at 20°C. After centrifugation at 10 Krpm for 5 min at room temperature, 200 μL of supernatant were withdrawn from each tube and diluted with 600 μL of H_2_O. The absorbance at 260 nM of each diluted sample was measured in a UV-1601 Shimadzu Spectrophotometer.
(JPG)

S1 Dataset Raw data of Fig 3. (XLSX)

S2 Dataset Raw and normalized data of Fig 5. (XLSX)

S3 Dataset Raw and normalized data of Fig 6. (XLSX)

S4 Dataset Raw and normalized data of Fig 7. (XLSX)
