# Supplementary figures and images for: Development of a graphene oxide-based assay for the sequence-specific detection of double-stranded DNA molecules
Source: PLoS One. 2017 Aug 29;12(8):e0183952. doi: 10.1371/journal.pone.0183952 (PMC5574608; doi:10.1371/journal.pone.0183952)

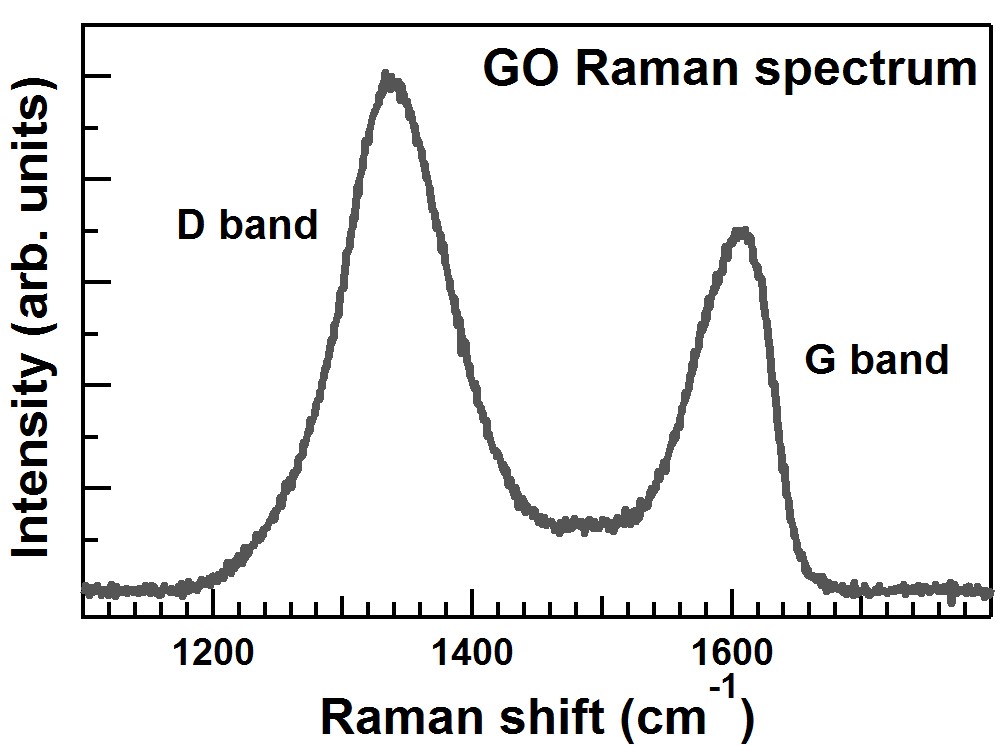

Supplement: S1 Fig — The GO Raman spectrum is characterized by the D and G band positioned respectively at 1330 and 1600 cm-1 and with a full width at half maximum of 100 and 74 cm-1. The relative intensity ID/IG is equal approximately to 1.4. (JPG) [file pone.0183952.s001.jpg]

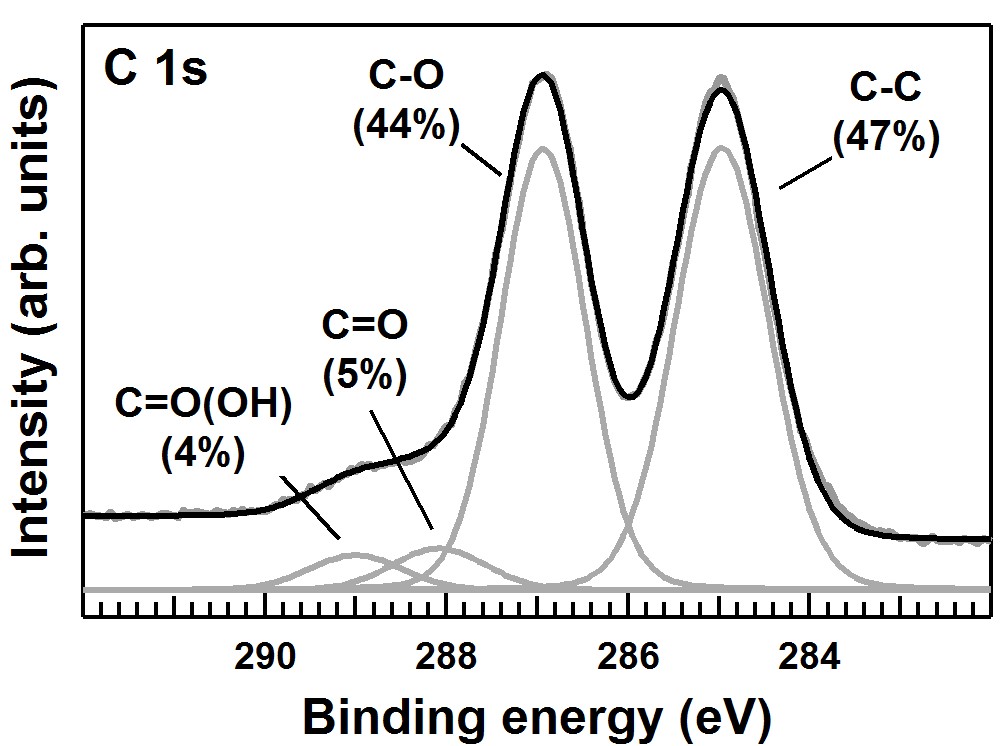

Supplement: S2 Fig — The C 1s spectrum is fitted by the sum of three components assigned to C atoms belonging to: aromatic rings and hydrogenated carbon (C = C/C-C, C-H, 284.9 eV), hydroxyl groups and epoxy groups (C-OH, C-O-C, 286.9 eV), carbonyl groups (C = O, 288.0) and carboxyl groups (C = O(OH), 289.0 eV). The relative weight of each component is equal to 47%, 44%, 5% and 4%, respectively, while the overall C/O ratio is ≈2. (JPG) [file pone.0183952.s002.jpg]

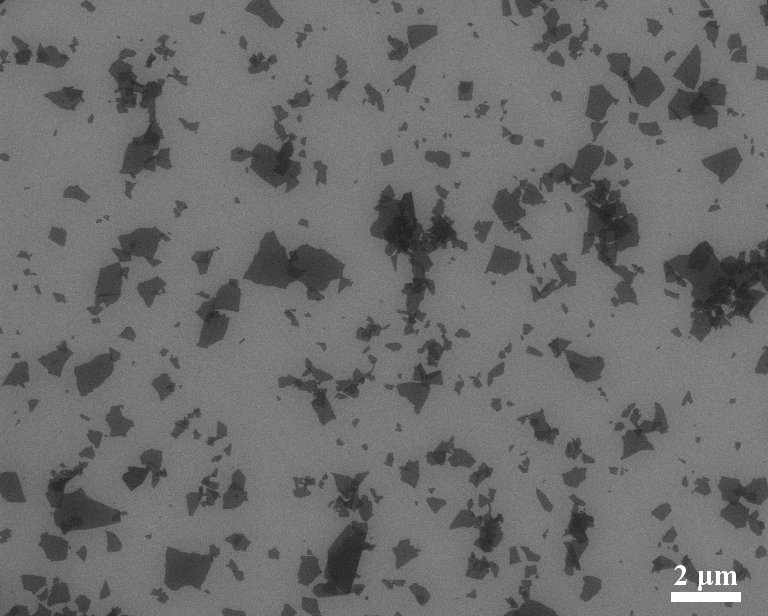

Supplement: S3 Fig — The image shows that the GO flakes size ranges between 0.2 and 2 μm. (JPG) [file pone.0183952.s003.jpg]

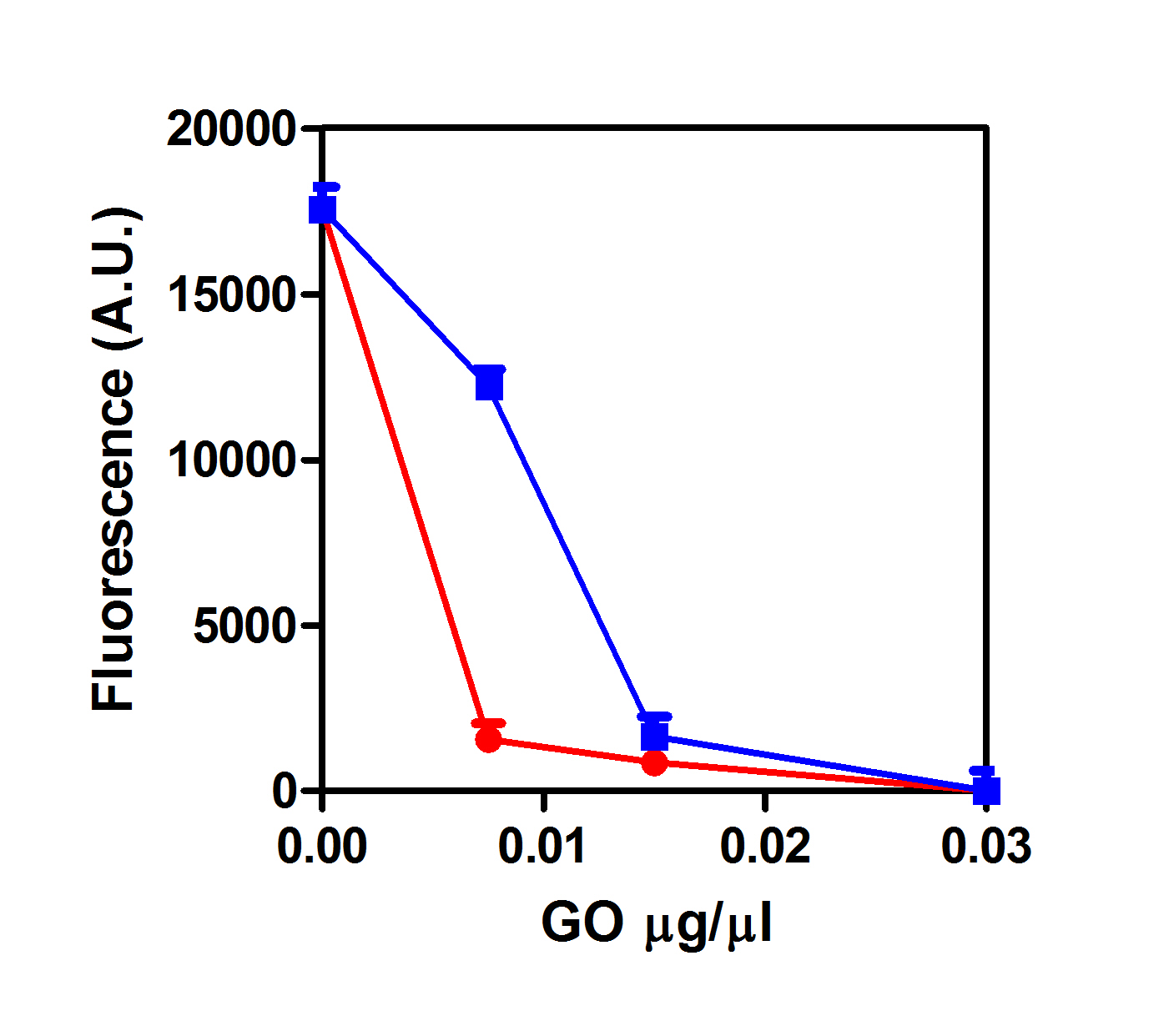

Supplement: S4 Fig — Further details are given in the text. The data points are the average of two experimental samples; error bars represent the standard deviations. The signal measured in the absence of the fluorophore was taken as background and subtracted from each experimental point. The percentage was calculated taking F-F0 as 100%, where F and F0 are the fluorescences measured in the presence and in the absence of the fluorescent primer, respectively. (JPG) [file pone.0183952.s004.jpg]

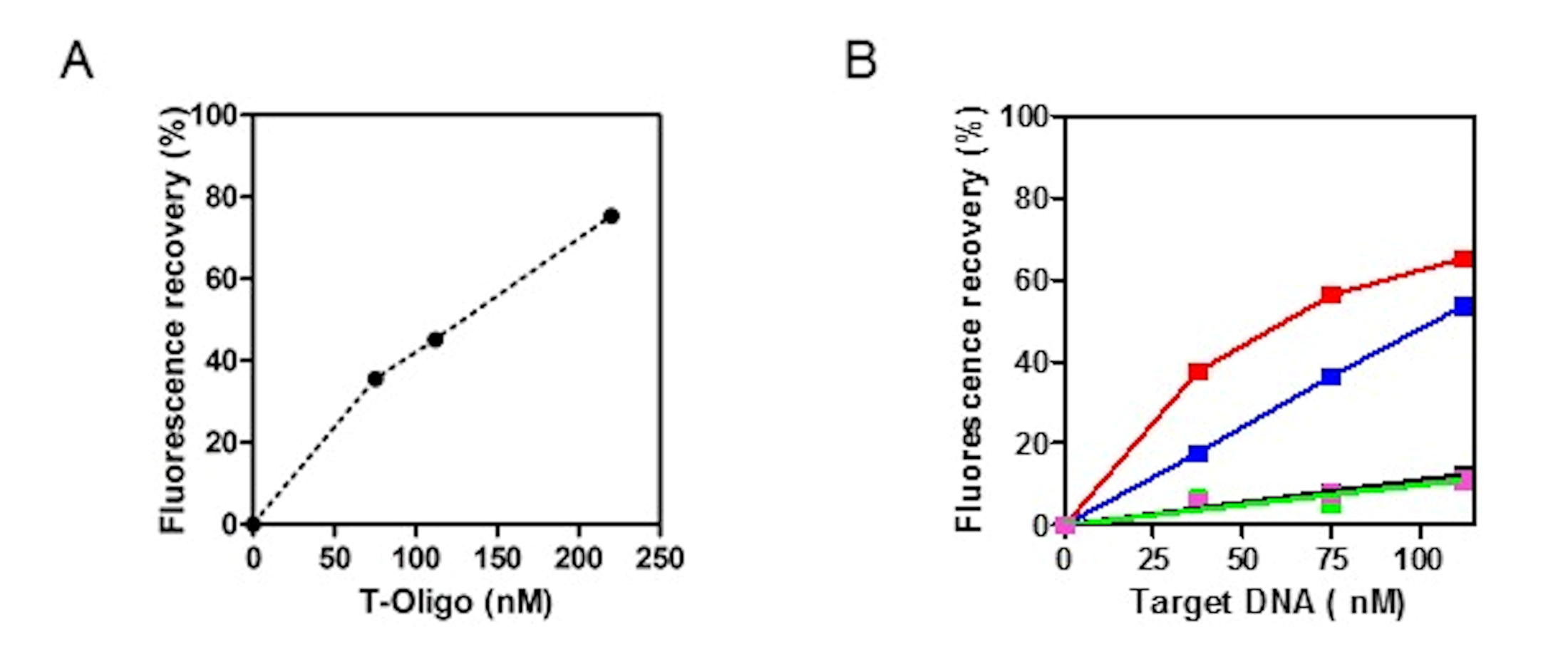

Supplement: S5 Fig — Relative fluorescence of FAM-P (75 nM) after incubation with the indicated concentrations of T-Oligo (A) or Target DNAs (B) and subsequent addition of GO (8 μg/ml). The target DNAs are: cspC (red square), cspE (blue triangle), cspA (magenta circle), cspB (open square) and hupA (green diamond). Further details are given in the text and in Materials and Methods. The signal measured with FAM-P +GO was taken as background and subtracted from each experimental point. The percentage is calculated taking F-FGO as 100%, where F and FGO are the fluorescences measured with FAM-P alone and FAM-P +GO, respectively. (TIF) [file pone.0183952.s005.tif]

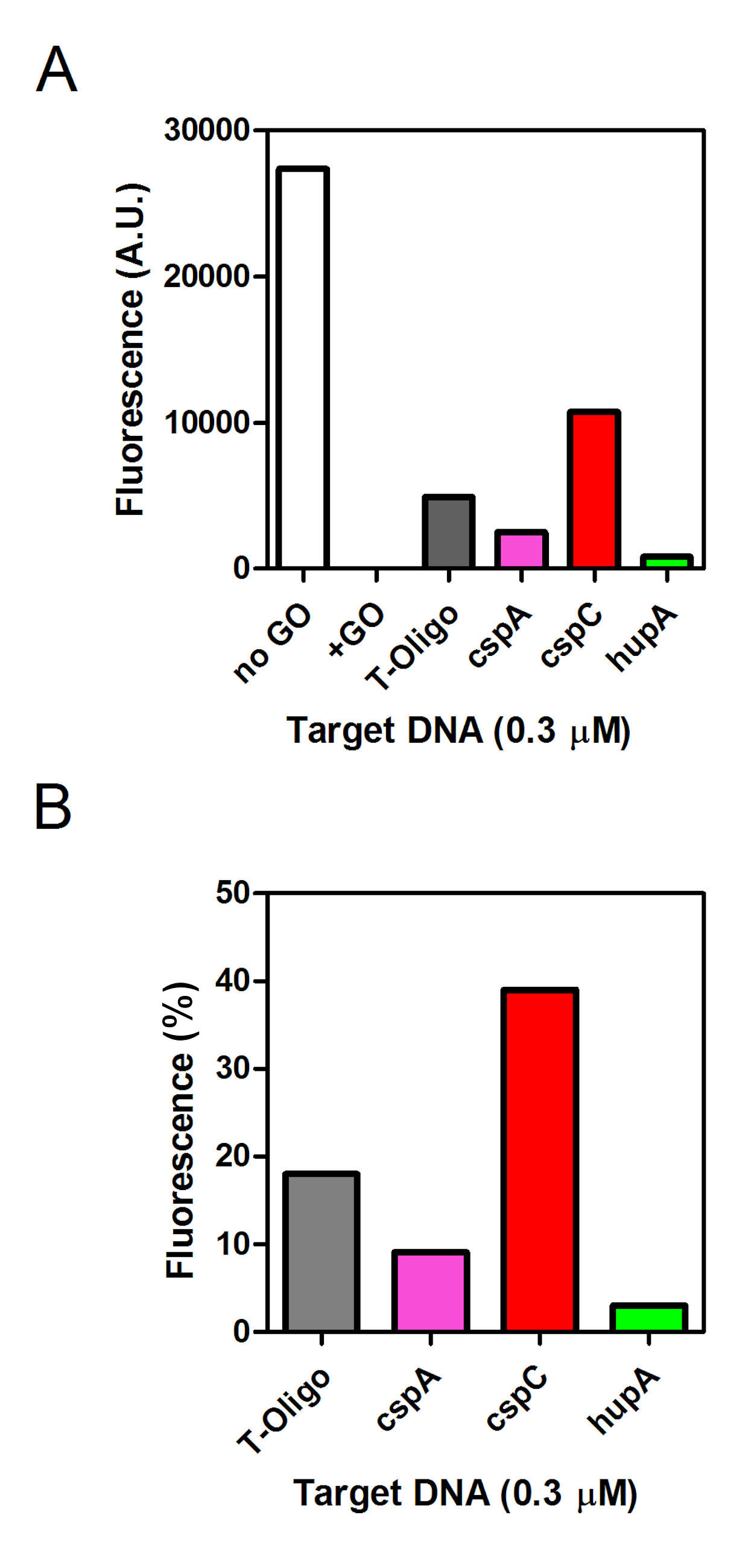

Supplement: S6 Fig — Absolute (A) or Relative (B) fluorescence of FAM-P (450 nM) after incubation with T-Oligo (450 nM) or the indicated Target DNAs (300 nM), and subsequent addition of GO (8 μg/ml). Further details are given in Material and Methods. The florescence measured with FAM-P +GO was taken as the background and subtracted from each experimental point. In (b), the percentage is calculated taking F-FGO as 100%, where F and FGO are the florescence measured with FAM-P alone and FAM-P +GO, respectively. (JPG) [file pone.0183952.s006.jpg]

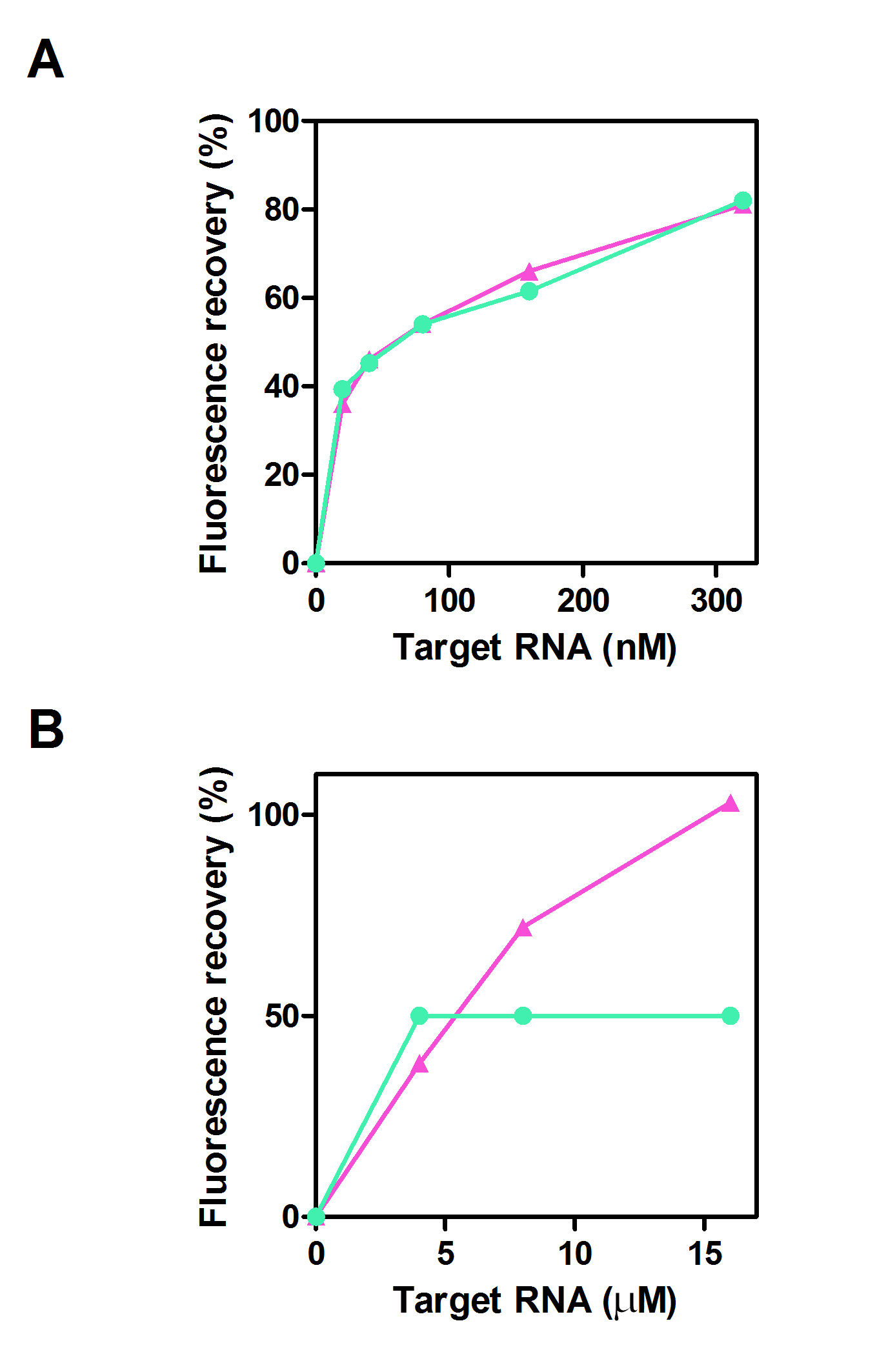

Supplement: S7 Fig — FAM-P at the concentration of (A) 75 nM or (B) 3 μM was incubated with the indicated concentration of cspA (magenta) or infA (cyano) mRNAs. The fluorescence was recorded after GO addition as described in Materials and Methods. The signal measured with FAM-P +GO was taken as background and subtracted from each experimental point. The percentage is calculated taking F-FGO as 100%, where F and FGO are the florescence measured with FAM-P alone and FAM-P +GO, respectively. (JPG) [file pone.0183952.s007.jpg]

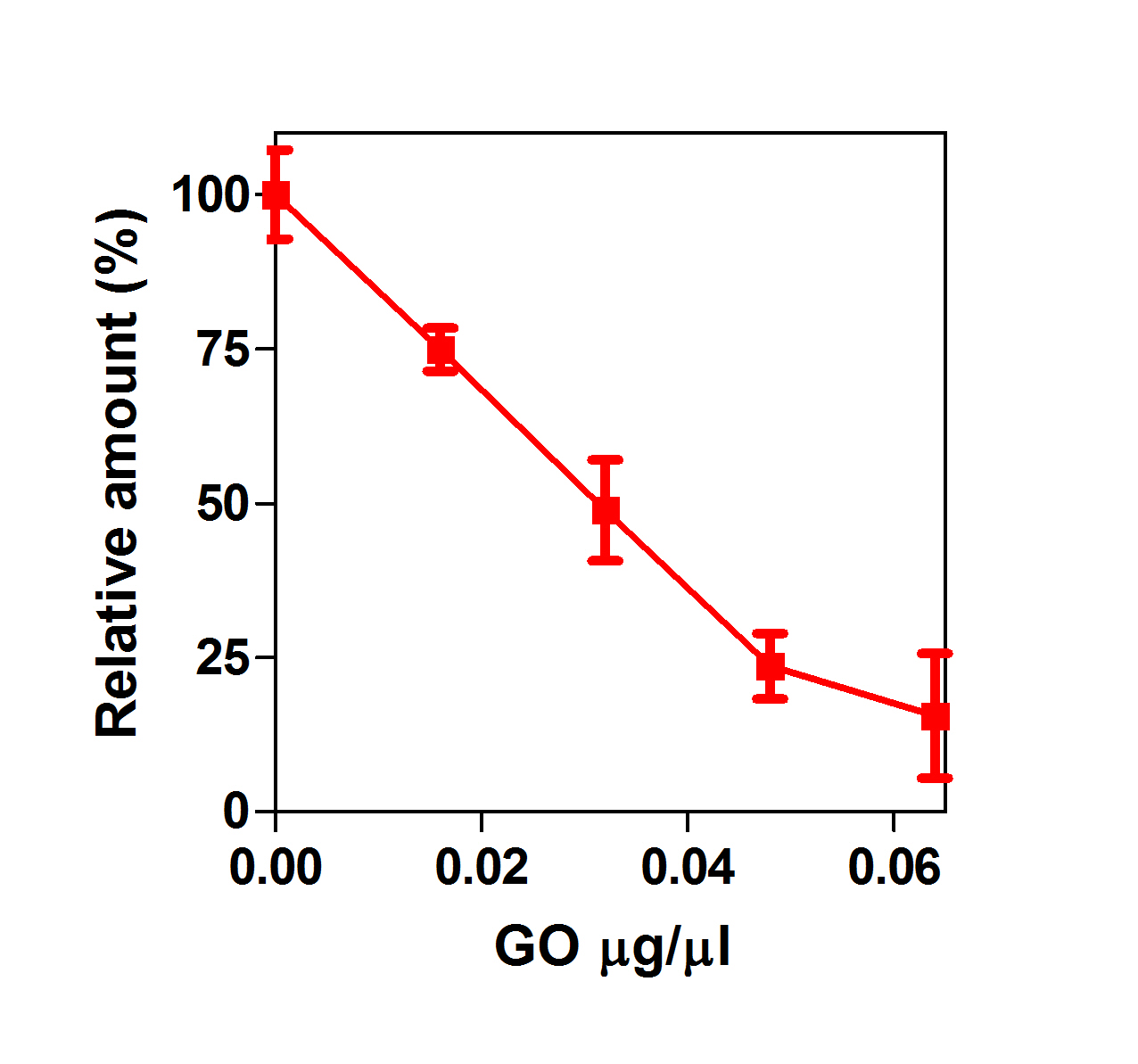

Supplement: S8 Fig — A fluorescent primer (24 pmoles) was incubated with the amounts of GO indicated in the figure under the conditions described in Materials and Methods. After centrifugation at 10 Krpm for 5 minutes, the fluorescence of 10 μL supernatants was read using the FLUOstar Omega instrument. The data points are the average of two experimental samples; error bars represent the standard deviations. The signal measured in the absence of the fluorophore was taken as background and subtracted from each experimental point. The percentage was calculated taking F-F0 as 100%, where F and F0 are the fluorescences measured in the presence and in the absence of the fluorescent primer, respectively. (JPG) [file pone.0183952.s008.jpg]

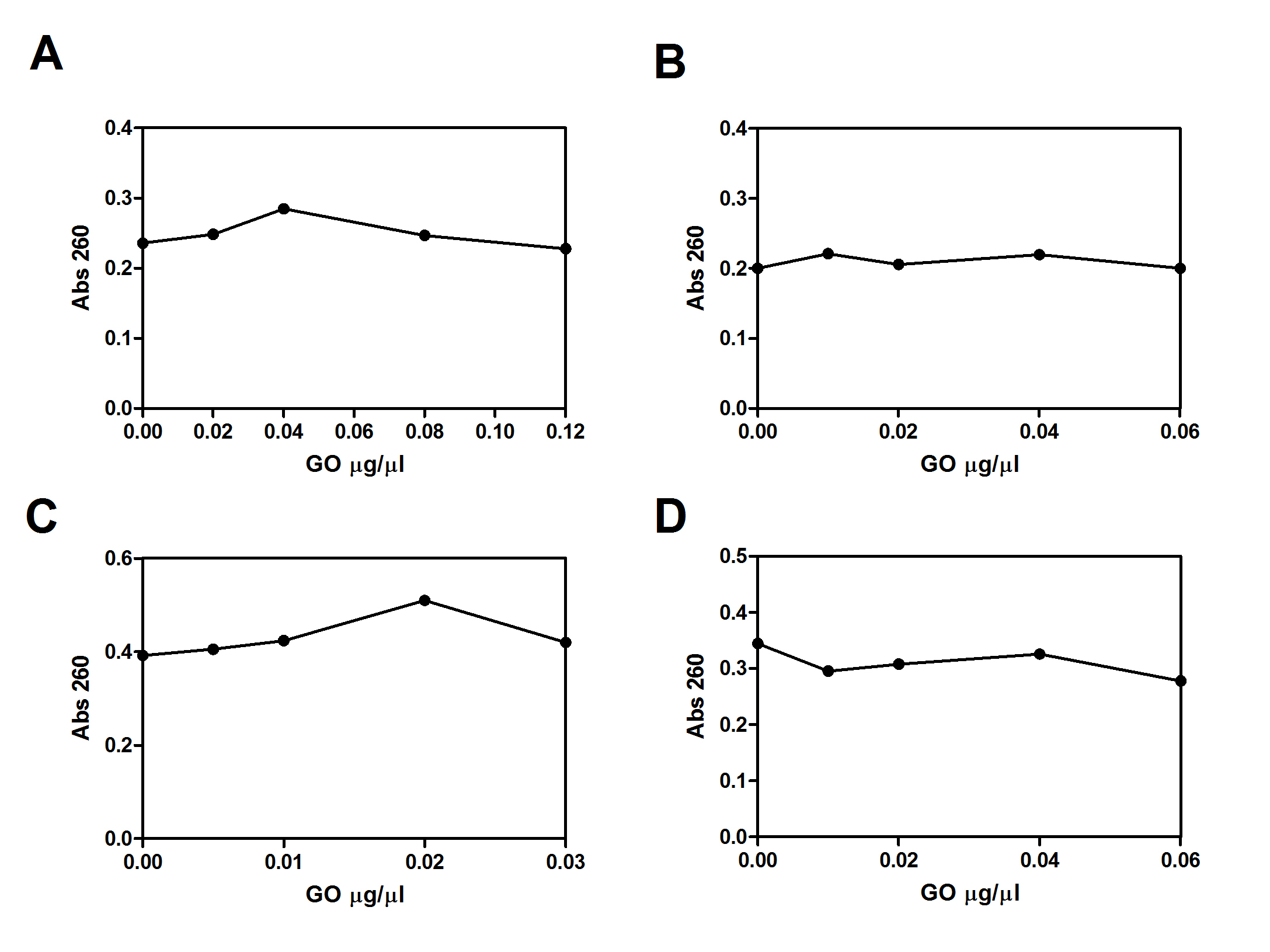

Supplement: S9 Fig — The GO amounts indicated in the figure were incubated in 1X GO buffer with (A) 60 μM and (B) 120 μM ATP or (C) 5 μM and (D) 160 μM Adenosine in 500 μL of reaction volume for 10 min at 20°C. After centrifugation at 10 Krpm for 5 min at room temperature, 200 μL of supernatant were withdrawn from each tube and diluted with 600 μL of H2O. The absorbance at 260 nM of each diluted sample was measured in a UV-1601 Shimadzu Spectrophotometer. (JPG) [file pone.0183952.s009.jpg]
